# Aberrant Expression of Collagen Gene Family in the Brain Regions of Male Mice with Behavioral Psychopathologies Induced by Chronic Agonistic Interactions

**DOI:** 10.1155/2019/7276389

**Published:** 2019-04-28

**Authors:** Dmitry A. Smagin, Anna G. Galyamina, Irina L. Kovalenko, Vladimir N. Babenko, Natalia N. Kudryavtseva

**Affiliations:** Laboratory of Neuropathology Modeling, Neurogenetics of Social Behavior Sector, Institute of Cytology and Genetics, Siberian Branch of Russian Academy of Sciences, Novosibirsk, Russia

## Abstract

Chronic agonistic interactions promote the development of experimental psychopathologies in animals: a depression-like state in chronically defeated mice and the pathology of aggressive behavior in the mice with repeated wins. The abundant research data indicate that such psychopathological states are associated with significant molecular and cellular changes in the brain. This paper aims to study the influence of a 20-day period of agonistic interactions on the expression patterns of collagen family genes encoding the proteins which are basic components of extracellular matrix (ECM) in different brain regions of mice using the RNA-Seq database. Most of differentially expressed collagen genes were shown to be upregulated in the hypothalamus and striatum of chronically aggressive and defeated mice and in the hippocampus of defeated mice, whereas downregulation of collagen genes was demonstrated in the ventral tegmental areas in both experimental groups. Aberrant expression of collagen genes induced by chronic agonistic interactions may be indicative of specific ECM defects in the brain regions of mice with alternative social experience. This is the first study demonstrating remodeling of ECM under the development of experimental disorders.

## 1. Introduction

The collagen gene family encodes collagen proteins which are the predominant glycoproteins of the extracellular matrix (ECM) and major structural elements of connective tissues in mammals. Collagens are fibrous proteins that build a framework of connective tissue, in particular, in skin, bones, tendons, cartilage, blood vessels, and teeth of vertebrates.

Collagens form fibrils that assemble into elongated collagen fibers. According to the literature data, so far, 28 collagen types and 45 genuine collagen encoding genes as well as genes coding the proteins containing a collagen-like domain have been identified [1–5]. Collagens play an important and multifunctional role in peripheral and central nervous systems (CNS) [6]. It is supposed, for example, that collagen XVI acts as an adaptor protein connecting and organizing large fibrillar networks and thus modulates integrity and stability of the ECM [7]. Collagen VI is key matrix component found in all connective tissues, where it forms abundant and structurally unique microfibrils in association with basement membranes and supports spreading, adhesion, migration, and survival of the cells [8–10]. Collagen VI-related myopathy, caused by mutations in the genes, encoding collagen protein type VI, is a group of disorders that affect skeletal muscles and connective tissue [11, 12]. Most affected individuals have muscle weakness and joint deformities called contractures that restrict movement of the affected joints and worsen over time. Fibrillar collagens have also been studied in relation to neurological diseases such as motor neuron diseases or amyotrophic lateral sclerosis [13, 14], indicating collagen metabolism alterations.

It is believed that fibrillar collagens are absent in the brain tissue, which contains collagens as structural components of the extracellular matrix, which are involved in axonal guidance, synaptogenesis, cell adhesion, the development of brain architecture,* etc.* [15–17]. Collagens play a major role in neural maturation [4, 18].

Some of the brain collagen proteins are expressed by neurons [19], suggesting their involvement in regulation of axonal outgrowth and synaptic differentiation [20]. According to recent study [21], the* Col4a1* and* Col4a2* genes are expressed by the endothelial cells, the* Col6a2, Col24a1, *and* Col25a1* genes are expressed by the neurons, and the* Col1a1 *and* Col3a1* genes are expressed by the oligodendrocyte precursor in neocortex. Collagen XIX is expressed by interneurons and contributes to the formation of hippocampal synapses [22]. It has been supposed that enhanced expression of the ECM related with cell adhesion genes, the* Col8a1* in particular, in the prefrontal cortex may affect cortical neural plasticity, including morphological neuronal changes and the afferent and/or efferent neural pathways participating in stress-related emotional behavioral patterns [23].

Biosynthesis of collagens in brain may be abnormal in many hereditary diseases, including the so-called collagen diseases. Most collagens-associated brain pathologies are related to neurodevelopment (collagens I, IV, and XVIII). Collagen type IV is known to inhibit glial differentiation in cortical cell cultures [24] and was increased in the frontal and temporal cortex of Alzheimer's disease patients. Significant ECM changes occur during the early stages of this disease [25]. There is genetic association to the amyloid plaque associated protein COL25A1 in Alzheimer's disease [26]. In these patients, extracellular collagenous Alzheimer amyloid plaque component [27], which is extracellular part of the transmembrane collagen XXV preferentially, is expressed in neurons: in other cell types its expression is observed as well, but at least 5-fold lower than in neurons [21].

Postmortem human studies showed that collagen XVII is widely expressed in the brain and is located primarily in the soma and proximal axons of neurons in contrast to glial cells, which do not express this collagen [28, 29]. Collagen XVI may act as a substrate for adhesion and invasion of connective tissue tumor cells. Alteration of tissue location and expression level appears to promote tumorigenesis and inflammatory reactions [7]. Thus, altering the cell-matrix interaction through collagen XVI might be a molecular mechanism to further augment the invasive phenotype of glioma cells.

Interestingly, amyloid-beta peptides increased mouse neuronal expression of collagen VI through a mechanism involving transforming growth factor signaling [30]. Overexpression of the* Col25a1 *gene in neurons of transgenic mice leads to Alzheimer's disease-like brain pathology [31]. Loss of interneuron-derived collagen XIX leads to a reduction in perineuronal nets in the mammalian telencephalon [32]. Genetic alterations in the region encoding collagen XIX and loss of this collagen in mice result in altered inhibitory synapses and in the acquisition of schizophrenia-related behaviors [33]. All these data indicate the possible role of collagen genes in different neurological disorders; however little is known about the reaction of brain collagen genes to chronic functional loading.

Chronic agonistic interactions in male mice induce the development of behavioral psychopathology characterized by mixed anxiety/depression-like states in defeated male mice [34–36], similar to those in humans, and psychosis-like state in male mice with repeated experience of aggression [37, 38]. It has been shown that under chronic social defeat stress the adult brain undergoes numerous molecular and cellular changes, including changes in gene expression in different brain regions [35, 39], DNA methylation, histone acetylation, and chromatin remodeling [40, 41], as well as decreases in hippocampal neurogenesis [42–44]. Changes in the expression of monoaminergic genes and increased hippocampal cell proliferation, as well as growth of new neurons, were previously found in male mice with repeated experience of aggression [45–48].

Additionally, we found the regions-specific changes of expression of most ribosomal* Rpl* and* Rps* genes and mitochondrial ribosomal* Mrpl* and* Mrps* genes [49, 50] as well as the* Slc25* genes encoding mitochondrial carrier proteins [51] in male mice with different social experience. All these data confirmed mitochondrial dysfunction in the pathological conditions induced by repeated agonistic interactions in mice. There has been demonstrated that mitochondria are involved in the pathogenesis of the muscular dystrophies [52]. Thus, the next step of our research was, using the same RNA-Seq database, to study the changes in the expression of collagen genes encoding the proteins which are basic components of the ECM in different brain regions of mice with experimental psychopathologies. Negative and positive social experience, social defeats and wins, were induced by 20-day period of agonistic interactions [34, 37]. The brain regions for testing were selected based on their functions, location of neurons of some neurotransmitter systems, and differential involvement in the mechanisms of social behaviors in our experimental paradigm.

## 2. Materials and Methods 

### 2.1. Animals

Adult male mice C57BL/6J were obtained from Animal Breeding Facility, Branch of Institute of Bioorganic Chemistry of the RAS (ABF BIBCh, RAS) (Pushchino, Moscow region). Animals were housed under standard conditions (12:12 hr light/dark regime starting at 8:00 am, at a constant temperature of 22+/-2°C, with food in pellets and water available* ad libitum*). Mice were weaned at three weeks of age and housed in groups of 8-10 in standard plastic cages, size 23 (width) × 36 (length) × 12 (height) cm. Experiments were performed with 10-12-week-old animals. All procedures were in compliance with the European Communities Council Directive 210/63/EU on September 22, 2010. The study was approved by Scientific Council N 9 of the Institute of Cytology and Genetics SD RAS of March, 24, 2010, N 613 (Novosibirsk).

### 2.2. Generation of Alternative Social Behaviors under Agonistic Interactions in Male Mice

Prolonged negative and positive social experience in male mice were induced by daily agonistic interactions [34, 37]. Pairs of weight-matched animals were each placed in a steel cage (14 × 28 x 10 cm) bisected by a perforated transparent partition allowing the animals to see, hear, and smell each other, but preventing physical contact. The animals were left undisturbed for two or three days to adapt to new housing conditions and sensory contact before they were exposed to encounters. Every afternoon (14:00-17:00 p.m. local time), the cage lid was replaced by a transparent one, and 5 min later (the period necessary for individuals' activation), the partition was removed for 10 minutes to encourage agonistic interactions. The superiority of one of the mice was firmly established within two or three encounters with the same opponent. The superior mouse would be attacking, biting, and chasing another, who would be displaying only defensive behavior (sideways postures, upright postures, withdrawal, lying on the back, or freezing). As a rule, aggressive interactions between males are discontinued by lowering the partition if the sustained attacks have lasted 3 min (in some cases less) thereby preventing the damage of defeated mice. Each defeated mouse (defeater, loser) was exposed to the same winner for three days, while afterwards each loser was placed, once a day after the fight, in an unfamiliar cage with an unfamiliar winner behind the partition. Each winning mouse (winners, aggressors) remained in its original cage. This procedure was performed once a day for 20 days and yielded an equal number of winners and losers.

Three groups of animals were used: (1) controls: mice without a consecutive experience of agonistic interactions; (2) losers: groups of chronically defeated mice after 20 days of agonistic interactions; (3) winners: groups of chronically aggressive mice. Twenty-day losers and winners with the most expressed behavioral phenotypes were selected for the transcriptome analysis. All mice were simultaneously decapitated, including winners and losers, 24 hours after the last agonistic interaction and the control animals. The brain regions were dissected by one experimenter according to the map presented in the Allen Mouse Brain Atlas [http://mouse.brain-map.org/static/atlas]. All biological samples were placed in RNAlater solution (Life Technologies, USA) and were stored at −70°C until sequencing.

The brain regions were selected for the analysis based on their functions and localization of neurons of neurotransmitter systems. These are as follows: the midbrain raphe nuclei, a multifunctional brain region, which contain the majority of serotonergic neuronal bodies; the ventral tegmental area (VTA), which contains the bodies of dopaminergic neurons, is widely implicated in natural reward circuitry of the brain, and is important in cognition, motivation, drug addiction, and emotions relating to several psychiatric disorders; the striatum, which is responsible for the regulation of motor activity and stereotypical behaviors and is also potentially involved in a variety of cognitive processes; the hippocampus, which belongs to the limbic system, is essential for memory consolidation and storage, and plays important roles in the neurogenesis and emotional mechanisms; and the hypothalamus, which regulates the stress reaction and many other physiological processes.

### 2.3. RNA-Seq

We used the RNA-Seq database described earlier in [49–51]. The collected samples were sequenced at JSC Genoanalytica (www.genoanalytica.ru, Moscow, Russia), and the mRNA was extracted using a Dynabeads mRNA Purification Kit (Ambion, Thermo Fisher Scientific, Waltham, MA, USA). cDNA libraries were constructed using the NEBNext mRNA Library PrepReagent Set for Illumina (New England Biolabs, Ipswich, MA USA) following the manufacturer's protocol and were subjected to Illumina sequencing. More than 20 million reads were obtained for each sample. The resulting “fastq” format files were used to align all reads to the GRCm38.p3 reference genome using the TopHat aligner [53]. DAVID Bioinformatics Resources 6.7 (http://david.abcc.ncifcrf.gov) was used for the description of differentially expressed gene ontology. The Cufflinks program was used to estimate the gene expression levels in FPKM units (fragments per kilobase of transcript per million mapped reads) units and subsequently identify the differentially expressed genes in the analyzed and control groups. Each brain area was considered separately for 3 versus 3 animals. Only annotated gene sequences were used in the following analysis. Genes were considered differentially expressed at* P ≤* 0.05 and q < 0.05 was also taken into consideration.

We have previously conducted studies of gene expression in males in similar experiments using the RT-PCR method with larger samples for each compared experimental group, i.e., winners and losers (>10 animals). The direction and extent of changes in expressions of the* Tph2, Slc6a4, Bdnf, Creb1*, and* Gapdh* genes in the midbrain raphe nuclei of males compared with the control produced by two methods, including RT-PCR and RNA-Seq, are generally consistent as was described in detail [49–51]. In order to cross-validate the results obtained, we employed the unique resource from Stanford University, USA [21], and found a significant concordance with our RNA-Seq data pool [54]. These findings suggest that the transcriptome analyses of the data provided by the ZAO Genoanalitika (http://genoanalytica.ru, Moscow) have been verified and that the method reflects the real processes that occur in the brain under our experimental paradigm.

The Human Gene Database (http://www.genecards.org/); Online Mendelian Inheritance in Man database (OMIM, http://omim.org/); Human disease database (MalaСards, http://www.malacards.org) were used for the description and analysis of the data obtained.

### 2.4. Statistical Analysis

For the transcriptomic data, a Principal components** (**PC) analysis was conducted using the XLStat software package (www.xlstat.com). It was based on a Pearson correlation metric calculated on the FPKM value profiles of 49 analyzed genes. Agglomerative Hierarchical Clustering (AHC) was performed on the same data with the XLStat software package. We also used a Pearson correlation as a similarity metric for the AHC analysis. The agglomeration method comprised an unweighted pair-group average.

## 3. Results and Discussion

The RNA-Seq data in FPKM units were processed for collagen related genes: 28 types of 45 genuine collagen genes; collagen-modifying enzymes genes (*Col4a3bp, Colgalt1, Colgalt2)*; collagen-like molecules* Adipoq, Ccbe1, Cthrc1, Colq, Colec11, Colec12, *and the* Adamts2, Bmp1, P4ha1, P4ha2, P4ha3, Pcolce, Pcolce2, Plod1, Plod2, *and* Plod3* genes encoding enzymes that modify procollagen molecules. The list of analyzed genes is given in S1 Table 1; FPKM units for samples are presented in S2 Table 1.

Whole transcriptome data analysis in the hypothalamus, midbrain raphe nuclei, hippocampus, ventral tegmental area (VTA), and striatum reveals similar and different changes in the expression of collagen (*Col*) genes in the brain regions of male mice after 20 days of agonistic interactions (Table 1). Most of* Col* genes that changed their expression were found in the hypothalamus (19 genes in both groups), hippocampus (15 genes in the losers), and striatum (19 genes in the losers). The minority of differentially expressed* Col* genes were found in the midbrain raphe nuclei (6 and 7 genes in the winners and losers, respectively), hippocampus and striatum (in total 5 genes in the winners), and VTA (11 and 7 genes in the winners and losers, respectively). Most of differentially expressed* Col *genes in the hypothalamus of the winners and losers as well as in the striatum and hippocampus of the losers were upregulated. In the VTA these genes were downregulated in the mice of both groups. (The full list of differentially expressed genes in different brain regions of the winners and losers as well as additional statistics are given in S1 Tables 2-4 and in the “Results” section.)

**Figure 1:**
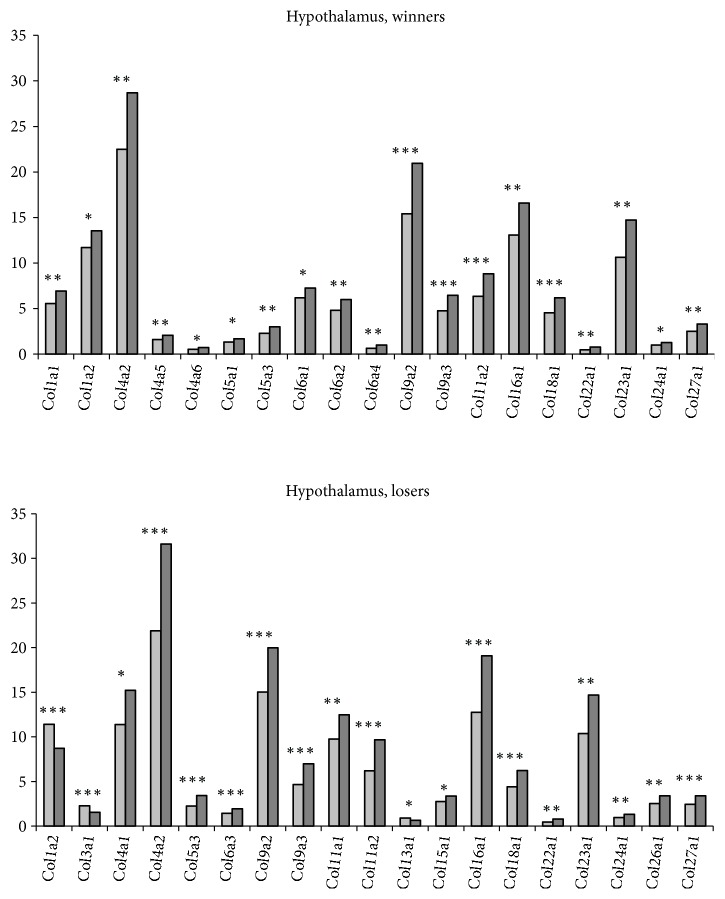
*The differentially expressed Col genes in the hypothalamus of mice with agonistic interactions.* The Cufflinks program was used to estimate the gene expression levels in FPKM units. The levels of the* Col *gene expression are presented in the control (left columns) and experimental mice (right columns) at the statistical significance: *∗ P* < 0.05; *∗∗P* < 0.01; and *∗∗∗P* < 0.001.

*In the hypothalamus* differentially expressed* Col4a2, Col5a3, Col9a2, Col9a3, Col11a2, Col16a1, Col18a1, Col22a1, Col23a1, Col24a1, Col27a1,* and* Plod3 *genes were upregulated in both social groups (Figure 1, S1 Tables 2 and 3, additional statistics). The* Col4a1, Col6a3, Col11a1, Col15a1, Col26a1, and Plod1 *genes were upregulated and* Col3a1, Col13a1, *and* Plod2 *genes were downregulated specifically in the losers. The* Col1a1, Col4a5, Col4a6, Col5a1, Col6a1, Col6a2, Col6a4, *and* Pcolce* genes were specifically upregulated in the winners. Expression of the* Col4a2, Col9a2*, and* Col16a1* genes and its changes were maximal in both social groups. The* Col1a2* gene was upregulated in the winners and downregulated in the losers.

The upregulation of most* Col* genes in the hypothalamus of the winners and losers may be response to chronic social stress inducing development of anxiety in both participants of social conflicts [55, 56]. Similar upregulation was previously found for most ribosomal* Rpl* and* Rps *genes and mitochondrial ribosomal* Mrpl∗* and* Mrps* genes in the hypothalamus of the winners and losers [49, 50]. It is therefore natural to assume that under chronic social stress of agonistic interactions many genes become coexpressed or jointly overexpressed.

The* Col1a2, Col3a1, *and* Col13a1 *genes were downregulated in the hypothalamus of the losers. According to GeneCards database the* Col1a2* and* Col3a1 *genes are associated with each other and both participate in the regulation of the vascular system. The function of the* Col13a1 *gene product, which is detected at low levels in all connective tissue-producing cells, is so far unknown. Unlike most collagens, which are secreted into the ECM, the* Col13a1-*encoded protein has been supposedly involved in cell-matrix and cell-cell adhesion interactions that are required for normal development. In contrast to the losers, the* Col1a2 *gene was upregulated in the winners, which may indicate that this gene is responsible for specific expression of the* Col* genes in the hypothalamus in each social group.

Little is known about the specific role that the collagen genes may play in the brain regions under functional loading. It was previously shown that the expression of* Col1a1* and C*ol3a1* genes was definitely observed in the pituitary glands of rats in the cells surrounding the capillaries, which are supposed to be the basic components of ECM [57]. Interestingly, similar to our results, the transcriptome analysis of hypothalamic gene expression during daily torpor in Djungarian hamsters revealed upregulation of the* Col18a1, Col5a3, Col17a1, *and* Col20a1* genes, which was specific for torpor as compared to normothermic hamsters [58]. Based on these data, one might preliminarily have hypothesized that the* Col* genes are involved in natural regulation, for example, of physiological adaptation under repeated stress in mice, winter torpor in hamsters, or CNS pathologies.

**Figure 2:**
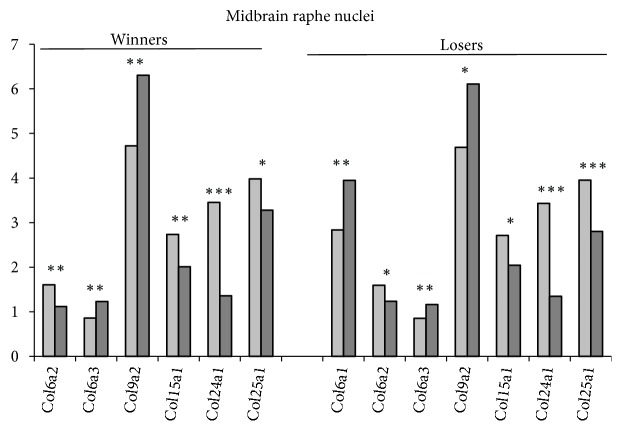
*The differentially expressed Col genes in the midbrain raphe nuclei of mice with repeated experience of agonistic interactions*. The Cufflinks program was used to estimate the gene expression levels in FPKM units. The levels of the* Col *gene expression are presented in the control (left columns) and experimental mice (right columns) at the statistical significance: *∗ P* < 0.05; *∗∗P* < 0.01; and *∗∗∗P* < 0.001.

*In the midbrain raphe nuclei* (Figure 2; S1 Tables 2 and 4; additional statistics) six* Col* genes in the winners and eight* Col *genes in the losers demonstrated modified expression under agonistic interactions. Expression of most genes therein was the lowest as compared with other regions (< 5.0 FPKM units). Nevertheless, similar to the hypothalamus, expression of the* Col9a2* gene was upregulated as compared to other genes. The* Col6a2, Col15a1, Col24a1, *and* Col25a1* genes were downregulated and the* Col6a3 *and* Col9a2* genes were upregulated similarly in both social groups. Specific alteration was upregulation of the* Col6a1, Ccbe1,* and* Colq* genes in the losers. Downregulation of the* Plod1* and* Plod3* genes and upregulation of the* Plod2* gene were specific for the winners (S1 Tables 2, 4).

The midbrain raphe nuclei contain the bodies of serotonergic neurons, which are involved in the regulation of many physiological, behavioral, and emotional processes. Repeated experience of aggression and defeats induced the decrease of serotonergic activity, which was accompanied by decreased expression of serotonergic genes such as* Tph2, Maoa, Slc6a4*, and* Htr*'s genes [39, 47] in this brain region.

It has been shown that stimulation of serotonin production may enhance the production of some collagen proteins, at least in human mesangial cells [59]. On the other hand, serotonin-dependent decrease in collagen mRNA was accompanied by decreased serotonin transcription downregulating the gene encoding type I collagen and other ECM proteins in myometrial cells [60]. Thus, it could be suggested that a similar decrease in* Col* gene expression in the midbrain raphe nuclei of the winners and losers can be associated with decreased serotonergic activity. The preliminary hypothesis, stemming from these data, may indicate the link between the downregulation of collagen genes and the reduced expression of serotonergic genes, shown earlier at least in this brain area.

**Figure 3:**
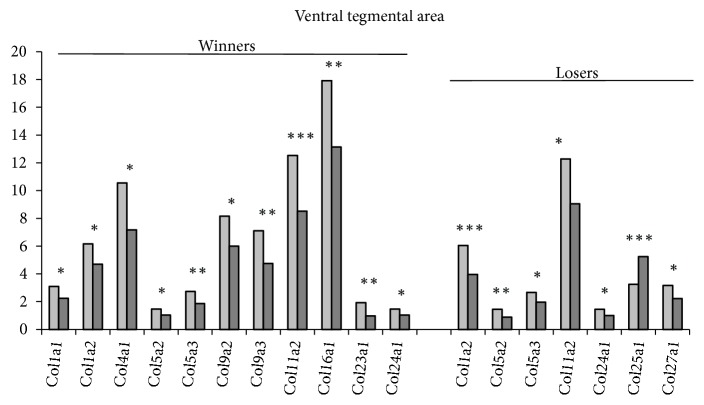
*The differentially expressed Col genes in the ventral tegmental area of mice with repeated experience of agonistic interactions*. The Cufflinks program was used to estimate the gene expression levels in FPKM units. The levels of the* Col *gene expression are presented in the control (left columns) and experimental mice (right columns) at the statistical significance: *∗ P* < 0.05; *∗∗P* < 0.01; and *∗∗∗P* < 0.001.

*In the VTA* (Figure 3, S1 Tables 2 and 4, additional statistics), twelve* Col* genes in the winners and nine genes in the losers altered their expression under agonistic interactions. All genes except the* Col25a1* and* Colgalt2* genes in the losers were downregulated in both social groups: the* Col1a2, Col5a2, Col5a3, Col11a2, Col24a1,* and* Colq* genes had lowered expression similarly in the winners and losers under agonistic interactions. The* Col1a1, Col4a1, Col9a2, Col9a3, Col16a1*, and* Col23a1 *genes specifically in the winners and the* Col27a1 *specifically in the losers were downregulated. Maximal expression in the control was observed for the* Col4a1, Col11a2, *and* Col16a1 *genes.

Comparing the changes of* Col* gene expression in the VTA and midbrain raphe nuclei of mice with experience of agonistic interactions, we noticed that only the* Col24a1* gene showed similarly decreased expression in both brain regions and in both social groups. However, in most cases genes with altered expression were different among social groups. Expression of some genes, for example, the* Col9a2* gene in the winners and the* Col25a1* and* Colq* genes in the losers, changed in opposite directions in the midbrain raphe nuclei and the VTA. Therefore, different processes in the ECM in different brain regions could be due to differences in neurogenic environments. It is worth noting that the VTA and midbrain raphe nuclei contain neuron bodies. In the midbrain raphe nuclei serotonergic neurons prevail. In the VTA dopaminergic neurons amount for 50-70% [61–63], GABAergic neurons for about 30% [63], and glutamatergic neurons for about 2-3% [62, 64] of total neurons. It is likely that similar changes in* Col* genes expression (downregulation) in the VTA and midbrain raphe nuclei might be due to neuronal processes* per se*, which are not known yet.

**Figure 4:**
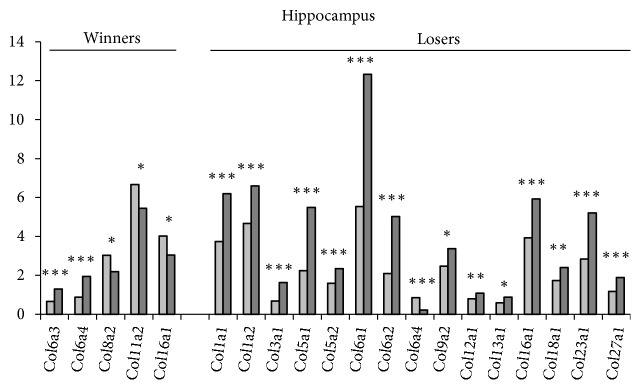
*The differentially expressed Col genes in the hippocampus of mice with repeated experience of agonistic interactions*. The Cufflinks program was used to estimate the gene expression levels in FPKM units. The levels of the* Col *gene expression are presented in the control (left columns) and experimental mice (right columns) at the statistical significance: *∗ P* < 0.05; *∗∗P* < 0.01; and *∗∗∗P* < 0.001.

*In the hippocampus* five* Col* genes in the winners and fifteen genes in the losers changed their expression under agonistic interactions (Table 1, Figure 4, S1 Tables 2 and 3; additional statistics). The* Col1a1, Col1a2, Col3a1, Col5a1, Col5a2, Col6a1, Col6a2, Col9a2, Col12a1, Col13a1, Col18a1, Col23a1, *and* Col27a1* genes were specifically upregulated in the losers and the* Col6a3* gene was specifically upregulated in the winners. Expression of the* Col11a2* and* Col8a2* genes was specifically downregulated in the winners. Interestingly, the expression of the* Col6a4* gene was increased in the winners and decreased in the losers. In these social groups expression of the* Col16a1* gene changed in opposite directions. Similar to the VTA, different changes in the ECM can be implied in male mice with alternative social behaviors.

Interestingly, in this region, all of the ribosomal genes were upregulated in the winners [50] and most of them were downregulated in the losers [49]. We suggested that the dynamic ribosomal gene expression patterns could be associated with differential cell proliferation in this brain region. Repeated aggression has been shown to be accompanied by an increase in the proliferation of neuronal progenitors and production of new neurons in the dentate gyrus of the hippocampus [48]. At the same time, other authors [42–44] have demonstrated decreased cell proliferation in this brain region of the losers. It was suggested that the changes in neurogenesis are the consequence of ribosomal dysfunction developing under chronic positive or negative social experience in daily agonistic interactions in our experimental paradigm. At present, we can suggest an association between the decreased neurogenesis, downregulation of ribosomal genes, and upregulation of the* Col* genes in the losers versus increased neurogenesis, upregulation of ribosomal genes, and downregulation of* Col* genes in the hippocampus of the winners. However, we cannot exclude the inverse relationship: changes in neurogenesis may influence the ribosomal and collagen genes functioning.

The link between neurogenesis and changes in* Col* gene expression was indirectly confirmed by the experiments [65]: oral administration of the lower molecular weight peptides derived from collagen enhanced hippocampal neurogenesis, exerted anxiety-related behavior in adult mice, and caused a 1.2-fold increase in the density of proliferating cells in the subgranular zone. We suggest that this model may be ideal for studying the mechanisms of neurogenesis activation and inhibition in the hippocampus.

**Figure 5:**
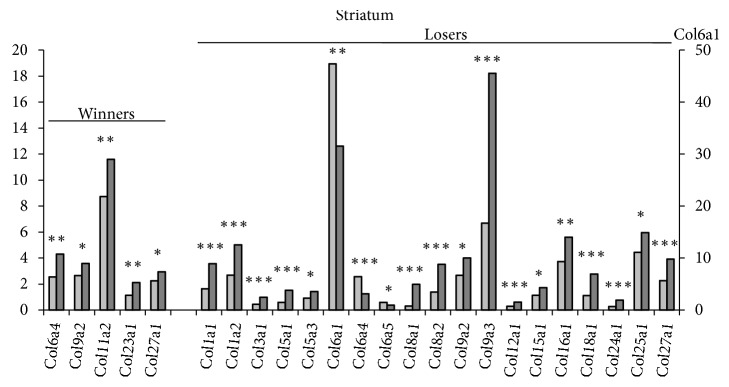
*The differentially expressed Col genes in the striatum of mice with repeated experience of agonistic interactions*. The Cufflinks program was used to estimate the gene expression levels in FPKM units. The levels of the* Col *gene expression are presented in the control (left columns) and experimental mice (right columns) at the statistical significance: *∗P* < 0.05; *∗∗P* < 0.01; and *∗∗∗P* < 0.001.

*In the striatum* (Table 1, Figure 5, S1 Tables 2 and 3; additional statistics), five* Col* genes in the winners and 20* Col *genes in the losers changed their expression under agonistic interactions. The* Col9a2* and* Col27a1* genes were upregulated in both social groups. In the winners, specific upregulation was shown for the* Col11a2* and* Col23a1* genes and in the losers for the* Col1a1, Col1a2, Col3a1, Col5a1, Col5a3, Col8a1, Col8a2, Col9a3, Col12a1, Col15a1, Col16a1, Col18a1, Col24a1, Col25a1, Colq, Cthrc1, Pcolce, Pcolce2, *and* Plod1 *genes, and downregulation was shown for the* Col6a1* and* Col6a5* genes. Similar to the hippocampus, the* Col6a4* gene was upregulated in the winners and downregulated in the losers. For the* Col6a1* gene, the highest expression in the control was shown in this region.

The striatum is responsible for the modulation of movement pathways and is potentially involved in a variety of other cognitive processes responsible for the regulation of motor activity and stereotypic behaviors. The winners demonstrate hyperactivity and enhanced locomotion in behavioral tests and stereotypical behaviors [37]. In this brain region of the losers with low locomotor and exploratory activities, helplessness, and immobility behavior in any situation, the total number of differentially expressed genes was 4 times higher than in the winners and the majority of such genes were upregulated [66]. Despite the differences in the locomotor behavior between the winners and losers, the majority of differentially expressed genes were upregulated in both social groups. Again we can suppose that differences in the types of* Col *genes involved in expression changes as well as the directions of such changes may depend on the neurogenic environment created by the leading neurotransmitter systems in the brain region. In the striatum 95% of all neurons are GABAergic [67] and, therefore, are closely connected to the glutamatergic system.

## 4. Principal Components Analysis of RNA-Seq* Col* Gene Expression Data

**Figure 6:**
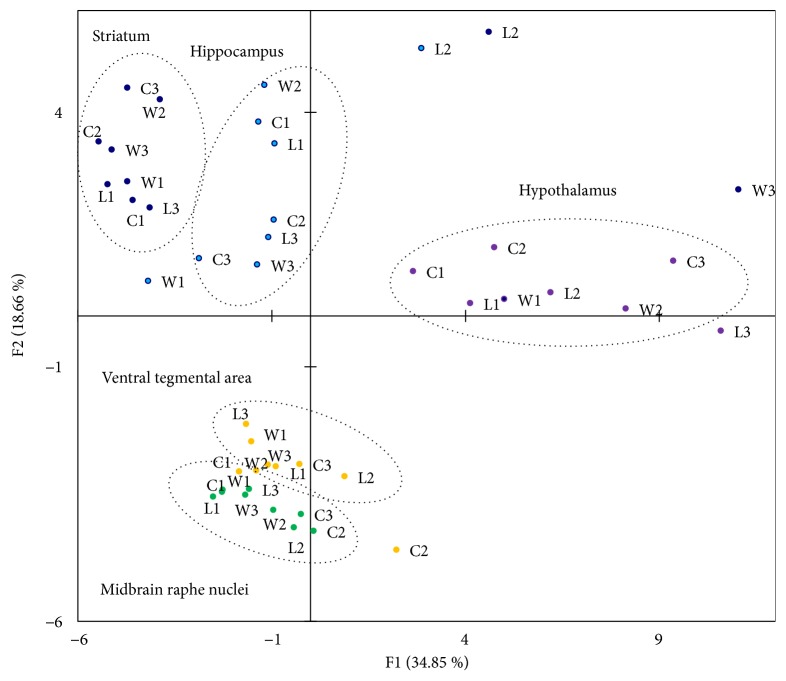
*Principal component analysis plot based on covariation of 49 Col genes using the expression profiles of 45 samples, which comprised RNA-Seq FPKM data for 5 brain regions of 9 mice*. Ovals correspond to brain regions. C1, C2, C3: control; W1, W2, W3: winners, aggressive mice; L1, L2, L3: defeated mice, losers. Distinct clustering of three brain regions occurred, whereas the midbrain raphe nuclei and ventral tegmental area were not distinct.

To assess the degree of brain region-specific expression of genes of interest, we performed a principal components analysis based on the covariation of genes using the expression profiles of 45 samples, which comprised RNA-Seq FPKM data for 5 brain regions of 9 mice. Ovals correspond to brain regions. We identified compact clustering of the hypothalamus, striatum, and hippocampus samples based on gene expression profiles (Figure 6, encircled), whereas the midbrain raphe nuclei and ventral tegmental area were closely located.

## 5. Agglomerative Hierarchical Clustering of the* Col* Genes Coding Transcripts and Identification of Outliers

**Figure 7:**
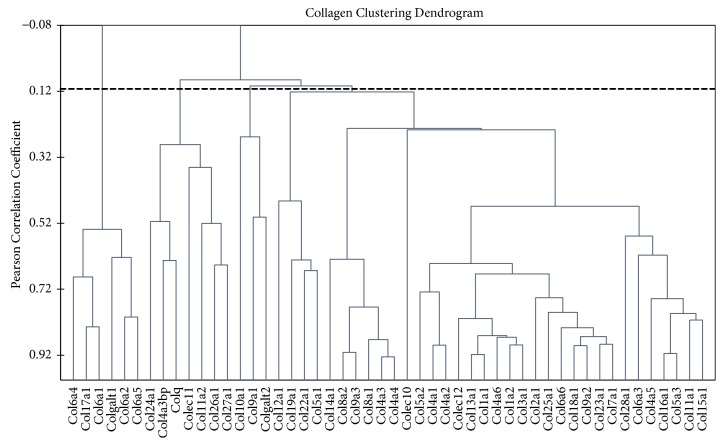
*Agglomerative hierarchical clustering of Col genes coding transcripts and identification of outliers.* We applied agglomerative hierarchical clustering to the initial sample of 49 genes across 45 samples, which comprised RNA-Seq FPKM data for 5 brain regions of 9 mice based on their expression profiles. Figure presents the dendrogram of gene clustering. The similarity ordinate corresponds to the Pearson correlation coefficient (df=44). Clusters differed at r = 0.1 (p<0.08) threshold.

We applied agglomerative hierarchical clustering (AHC) to the initial sample of 50 genes across 45 samples, which comprised RNA-Seq FPKM data for 5 brain regions of 9 mice based on their expression profiles (the dendrogram of gene clustering is presented in Figure 7). The similarity ordinate corresponds to the Pearson correlation coefficient (df=44). Using AHC analysis we identified tightly correlated genes, thereby strengthening the confidence in consistent differential expression of particular groups of genes based on their highly correlated expression profiles across 45 samples and particular brain regions (9 samples per region), as well as independent comparisons (disregarding the correlation) of expression rate for individual genes using the CuffDiff program. The overlapping or specific* Col* genes in different brain areas of the winners and losers are presented in S1 Tables 5, 6.

## 6. General Discussion

As for collagen genes, we would like to emphasize two main points. We previously confirmed the correlations of differentially expressed ribosomal and mitochondrial ribosomal genes for several brain regions in the winners and losers. Using the PC analysis, we also demonstrated consistent expression profiles for separate groups of collagen genes as well as regional specificity of gene expression (Figure 6). We found that most collagen genes form tightly correlated groups featuring their brain region specific expression (Figure 7). The corresponding groups were proved to be coordinated also by string-db network application (www.string-db.org).

The second point is that besides the brain region–specific expression pattern, we also observed an additional stress-mediated effect within certain brain regions resulting in the altered expression profile of collagen genes seen in animals. In the hypothalamus, striatum, and hippocampus most of the* Col* genes were upregulated, and the number of differentially expressed genes in the striatum and hippocampus was significantly higher in the losers than in the winners. In the midbrain raphe nuclei and VTA most* Col* genes were downregulated. It has been shown that the direction of changes in expression of some* Col* genes also depended on the social status of mice: for some genes changes in expression were either similar or specific among the winners and losers.

Our attention was drawn to the genes exhibiting inverse expression changes in the mice with different social experience. In the hypothalamus the* Col1a2* gene was upregulated in the winners and downregulated in the losers. In the hippocampus the* Col16a1* gene was downregulated in the winners and upregulated in the losers. In the striatum and hippocampus, the* Col6a4 *gene was upregulated in the winners and downregulated in the losers. The* Col6a4* gene was previously described in the brain and was shown to be expressed in the nervous system [30, 68] at low levels in different parts of the brain. We reconfirmed low expression of this gene in the brain regions.

Aberrant expression of the* Col* genes was demonstrated in the brain of male mice induced by a long experience of agonistic interactions. Since the* Col* genes encode collagen proteins, which are the predominant glycoproteins of the ECM and the main component of connective tissue in mammals [1–3], we can assume the ECM dysfunction induced by aberrant* Col* gene expression, which may be a consequence of altered neurotransmitter activities under repeated aggression or defeats or may be part of altered neurogenic environment exerting effect on major neurotransmitter systems which are involved in the regulation of pathological states. Analyzing the whole transcriptome in five brain regions of mice with mixed anxiety/depression state or in aggressive mice with psychopathology similar to psychosis we have found changes in the expression of serotonergic, dopaminergic, glutamatergic, and GABAergic genes. Presumably this is the altered neurotransmitter activity that creates primarily the conditions for the development of aberrant ECM functioning, which, as a consequence, produces changes in the operation of genes, including ribosomal and mitoribosomal genes, as previously shown for the brain regions [49, 50]. We can hypothesize that genes with inverse expression changes in the winners and losers are most sensitive to negative social factors that can generate the biogenesis of collagen abnormalities. Thus, there is a strong link between the experience of aggression or repeated defeats and brain region-specific changes in collagen gene expression. It can be suggested that neurogenic environment differs across the brain regions and that the specifics of metabolism emerging under repeated agonistic interaction can be mediated by modulation of collagen gene expression.

Collagens apparently can serve many purposes, such as to mediate cell adhesion, to segregate tissues from one another and regulate intercellular connection, to transmit signals to cell surface adhesion receptors, and to participate in the regulation of synaptic plasticity [69, 70]. Changes in physiological conditions can trigger rapid, local growth factor-mediated activation of cellular functions without additional synthesis. The stiffness and elasticity of the ECM appear to have important implications for cell migration, gene expression, and differentiation [71, 72]. The precise expression pattern depends on a balance of positive and negative transcription factors, proteins that control the synthesis of mRNA [73]. Authors suggest that developing or diseased nervous system is characterized by the expression of specific genes in order to make or repair the ECM. In any case, as previously hypothesized [74, 75] there are dynamic interrelations between the ECM and gene expression mediated by transmembrane proteins. Changes in the expression of numerous genes may in turn have an impact on cell interactions with other cells, creating a novel biogenic environment, which may be a cause of aberrant* Col* gene expression.

Most collagen disorders are associated with neurological abnormalities, including seizures and myoclonus, psychomotor retardation, spasticity, motor neuron disease, weakness, chronic fatigue, and endocrine abnormalities. It has been shown that aggressive and defeated mice show impaired motor activity and neurological symptoms [66] in the framework of developing psychopathologies induced by chronic agonistic interactions. This is the first study demonstrating remodeling of ECM under the development of experimental disorders.

It is well known that recurrent aggression and/or depression are a frequent symptom of many psychiatric and neurological disorders, including obsessive-compulsive and attention/deficit hyperactivity disorders, bipolar and posttraumatic stress disorders, epilepsy, mental retardation, autism, schizophrenia, and drug abuse [76]. Our experimental data indicate that all of these disorders may be accompanied by aberrant collagen functioning in the brain. However, it is to be mentioned that, on the one hand, changes in the expression of some collagen genes may be specific for the mouse brain. On the other hand, repeated experience of agonistic interaction may induce enormous brain regulation-mediated changes which may induce psycho- and neuropathologies, which* per se* may induce abnormalities in the ECM function.

At this stage of research, it is impossible to elucidate the detailed succession of neurochemical events and all molecular changes that occur as a result of changes in brain regulation in male mice under repeated agonistic interactions. However, it is clear that this process, starting with a change in social behaviors, at a certain stage, launches a cascade of systemic changes to the whole brain, specific regions, metabolism, neurons, and the neurotransmitter systems, which leads to altered expression of numerous genes, including collagen genes which encode a great part of the ECM biomolecules. Aberrant expression of collagen genes is likely to be accompanied by disruption of cell proliferation, translation, and transcription and is a consequence but not a cause of different psychopathologies induced by repeated agonistic interactions. The next stage of our work will be to find the key, major genes that run the entire chain of events from changes in social behaviors to changes in gene expression.

## Figures and Tables

**Table 1:** Differentially expressed *Col* genes in the brain regions of male mice with repeated experience of agonistic interactions (P < 0.05).

	MRN	HPC	VTA	STR	HPT
*Winners*					
All genes, N	6	5	11	5	19
Upregulation	2	2	0	5	19
Downregulation	4	3	11	0	0
*Losers*					
All genes, N	7	15	7	19	19
Upregulation	3	14	1	16	16
Downregulation	4	1	6	3	3

Note: MRN: midbrain raphe nuclei; HPC: hippocampus; VTA: ventral tegmental area; STR: striatum; HPT: hypothalamus.

## Data Availability

The additional statistics of data obtained used to support the findings of this study are available from Supplement 1 (Section Results and Tables 1-7) and Supplement 2 (Table 1, the* Col* genes expression in FPKM units) and are cited at relevant places within the text. The other datasets generated during the current study are available from the corresponding author on reasonable request.

## Supplementary Materials

Supplementary Materials Additional Files: Supplement 1 (S1): detailed description of differentially expressed* Col* genes specific for brain regions of male mice with repeated experience of agonistic interactions (Tables 1-6). S1 Table 1: list of analyzed* Col* genes.* S1 Results*: additional statistics for differentially expressed* Col* genes in brain regions of male mice with repeated experience of agonistic interactions. S1 Table 2: differentially expressed* Col* genes in brain regions of male mice with repeated experience of agonistic interactions. S1 Table 3: differentially expressed* Col* genes in the hippocampus, striatum, and hypothalamus of male mice with repeated experience of agonistic interactions. S1 Table 4: differentially expressed* Col* genes in the midbrain raphe nuclei and VTA in male mice with repeated experience of agonistic interactions. S1 Table 5: overlapping and specific differentially expressed genes in brain regions of mice with repeated experience of aggression. S1 Table 6: overlapping and specific differentially expressed genes in brain regions of mice with repeated experience of defeats. S1 Table 7: comparison of* Col* gene expression (FPKM units) in the winners* versus* losers. Supplement 2 (S2): S2 Table 1: FPKM values for 49* Col* transcripts analyzed in the study.
