# Supplementary material for: Aberrant Expression of Collagen Gene Family in the Brain Regions of Male Mice with Behavioral Psychopathologies Induced by Chronic Agonistic Interactions
Source: Biomed Res Int. 2019 Apr 28;2019:7276389. doi: 10.1155/2019/7276389 (PMC6512038; doi:10.1155/2019/7276389)
Supplement: Supplementary Materials — Additional Files: Supplement 1 (S1): detailed description of differentially expressed Col genes specific for brain regions of male mice with repeated experience of agonistic interactions (Tables 1-6). S1 Table 1: list of analyzed Col genes. S1 Results: additional statistics for differentially expressed Col genes in brain regions of male mice with repeated experience of agonistic interactions. S1 Table 2: differentially expressed Col genes in brain regions of male mice with repeated experience of agonistic interactions. S1 Table 3: differentially expressed Col genes in the hippocampus, striatum, and hypothalamus of male mice with repeated experience of agonistic interactions. S1 Table 4: differentially expressed Col genes in the midbrain raphe nuclei and VTA in male mice with repeated experience of agonistic interactions. S1 Table 5: overlapping and specific differentially expressed genes in brain regions of mice with repeated experience of aggression. S1 Table 6: overlapping and specific differentially expressed genes in brain regions of mice with repeated experience of defeats. S1 Table 7: comparison of Col gene expression (FPKM units) in the winners versus losers. Supplement 2 (S2): S2 Table 1: FPKM values for 49 Col transcripts analyzed in the study. [file 7276389.f1.zip › S1 Tables 1-7_BMRI_2744818.doc]

Smagin et al. Aberrant expression of collagen family genes in the brain regions of male mice with behavioral psychopathologies induced by chronic agonistic interactions

**Supplement 1 (S1). Detailed description of differentially expressed *Col** genes specific for brain regions of male mice with repeated experience of agonistic interactions (Tables 1-6), additional statistics.**

| **S1 Table 1. List of analyzed *Col* genes** | | |
| --- | --- | --- |
| *Col1a1* | | collagen, type I, alpha 1 (Col1a1), mRNA. |
| *Col1a2* | | collagen, type I, alpha 2 (Col1a2), mRNA. |
| *Col2a1* | | collagen, type II, alpha 1 (Col2a1), mRNA. |
| *Col3a1* | | collagen, type III, alpha 1 (Col3a1), mRNA. |
| *Col4a1* | | collagen, type IV, alpha 1 (Col4a1), mRNA. |
| *Col4a2* | | collagen, type IV, alpha 2 (Col4a2), mRNA. |
| *Col4a3* | | collagen, type IV, alpha 3 (Col4a3), mRNA. |
| *Col4a4* | | collagen, type IV, alpha 4 (Col4a4), mRNA. |
| *Col4a5* | | collagen, type IV, alpha 5 (Col4a5), mRNA. |
| *Col4a6* | | collagen, type IV, alpha 6 (Col4a6), mRNA. |
| *Col5a1* | | collagen, type V, alpha 1 (Col5a1), mRNA. |
| *Col5a2* | | collagen, type V, alpha 2 (Col5a2), mRNA. |
| *Col5a3* | | collagen, type V, alpha 3 (Col5a3), mRNA. |
| *Col6a1* | | collagen, type VI, alpha 1 (Col6a1), mRNA. |
| *Col6a2* | | collagen, type VI, alpha 2 (Col6a2), mRNA. |
| *Col6a3* | | collagen, type VI, alpha 3 (Col6a3), mRNA. |
| *Col6a4* | | collagen, type VI, alpha 4 (Col6a4), mRNA. |
| *Col6a5* | | collagen, type VI, alpha 5 (Col6a5), mRNA. |
| *Col6a6* | | collagen, type VI, alpha 6 (Col6a6), mRNA. |
| *Col7a1* | | collagen, type VII, alpha 1 (Col7a1), mRNA. |
| *Col8a1* | | collagen, type VIII, alpha 1 (Col8a1), mRNA. |
| *Col8a2* | | collagen, type VIII, alpha 2 (Col8a2), mRNA. |
| *Col9a1* | | collagen, type IX, alpha 1 (Col9a1), mRNA. |
| *Col9a2* | | collagen, type IX, alpha 2 (Col9a2), mRNA. |
| *Col9a3* | | collagen, type IX, alpha 3 (Col9a3), mRNA. |
| *Col10a1* | | collagen, type X, alpha 1 (Col10a1), mRNA. |
| *Col11a1* | | collagen, type XI, alpha 1 (Col11a1), mRNA. |
| *Col11a2* | | collagen, type XI, alpha 2 (Col11a2), mRNA. |
| *Col12a1* | | collagen, type XII, alpha 1 (Col12a1), mRNA. |
| *Col13a1* | | collagen, type XIII, alpha 1 (Col13a1), mRNA. |
| *Col14a1* | | collagen, type XIV, alpha 1 (Col14a1), mRNA |
| *Col15a1* | | collagen, type XV, alpha 1 (Col15a1), mRNA |
| *Col16a1* | | collagen, type XVI, alpha 1 (Col16a1), mRNA |
| *Col17a1* | | collagen, type XVII, alpha 1 (Col17a1), mRNA. |
| *Col18a1* | | collagen, type XVIII, alpha 1 (Col18a1), mRNA. |
| *Col19a1* | | collagen, type XIX, alpha 1 (Col19a1), mRNA. |
| *Col20a1* | | collagen, type XX, alpha1 (Col20a1), mRNA. |
| *Col21a1* | | collagen type XXI, alpha 1 (Col21a1), mRNA. |
| *Col22a1* | | collagen, type XXII, alpha 1 (Col22a1), mRNA. |
| **S1 Table 1.** Continuation | | |
| *Col23a1* | collagen, type XXIII, alpha 1 (Col23a1), mRNA. | |
| *Col24a1* | collagen, type XXIV, alpha 1 (Col24a1), mRNA. | |
| *Col25a1* | collagen, type XXV, alpha 1 (Col25a1), mRNA. | |
| *Col26a1* | collagen, type XXVI, alpha 1 (Col26a1), mRNA. | |
| *Col27a1* | collagen, type XXVII, alpha 1 (Col27a1), mRNA. | |
| *Col28a1* | collagen, type XXVIII, alpha 1 (Col28a1), mRNA. | |
|  |  | |
|  | **Collagen-modifying enzymes** | |
| *Col4a3bp* | collagen, type IV, alpha 3 binding protein (Col4a3bp), mRNA. | |
| *Colgalt1* | collagen beta(1-O)galactosyltransferase 1 (Colgalt1), mRNA. | |
| *Colgalt2* | collagen beta(1-O)galactosyltransferase 2 (Colgalt2), mRNA. | |
|  |  | |
|  | **Collagen-like molecules** | |
| *Adipoq* | adiponectin, C1Q and collagen domain containing (Adipoq), mRNA. | |
| *Ccbe1* | collagen and calcium binding EGF domains 1 (Ccbe1), mRNA. | |
| *Cthrc11* | collagen triple helix repeat containing 1 | |
| *Colq* | collagen-like tail subunit (single strand of homotrimer) of asymmetric acetylcholinesterase (Colq), mRNA. | |
| *Colec11* | collectin subfamily member 11 (Colec11*)*, collagen-like sequences | |
| *Colec12* | collectin subfamily member *12* (Colec12), collagen-like sequences | |
|  |  | |
|  | **Genes encoding enzymes that modify procollagen molecules** | |
| *Adamts2* | Procollagen N-Endopeptidase, 2(Adamts2) | |
| *Bmp1* | Procollagen C-Endopeptidase, [bone morphogenetic protein 1(Bmp1)](https://david.ncifcrf.gov/geneReportFull.jsp?rowids=12153) | |
| *P4ha1* | procollagen-proline, 2-oxoglutarate 4-dioxygenase (proline 4-hydroxylase), alpha 1 polypeptide (P4ha1), mRNA. | |
| *P4ha2* | procollagen-proline, 2-oxoglutarate 4-dioxygenase (proline 4-hydroxylase), alpha II polypeptide (P4ha2), transcript variant 1, mRNA. | |
| *P4ha3* | procollagen-proline, 2-oxoglutarate 4-dioxygenase (proline 4-hydroxylase), alpha polypeptide III (P4ha3), mRNA. | |
| *Plod1* | procollagen-lysine, 2-oxoglutarate 5-dioxygenase 1 (Plod1), mRNA. | |
| *Plod2* | procollagen lysine, 2-oxoglutarate 5-dioxygenase 2 (Plod2), transcript variant 2, mRNA. | |
| *Plod3* | procollagen-lysine, 2-oxoglutarate 5-dioxygenase 3 (Plod3), mRNA. | |
| *Pcolce* | procollagen C-endopeptidase enhancer protein (Pcolce), mRNA. | |
| *Pcolce2* | procollagen C-endopeptidase enhancer 2 (Pcolce2), mRNA. | |
|  |  | |

**Results**

**Additional statistics for differentially expressed *Col* genes in brain regions of male mice with repeated experience of agonistic interactions**

**In the hypothalamus** the *Col** genes increased their expression both in the winners and losers in comparison with the controls as for genes: *Col4a2* (*~~P~~* ~~≤ 0.003~~ *P* < 0.0032and *P* < 0.0001; q < 0.001, respectively)*, Col5a3* (*P* < 0.003; q < 0.047 and *P* < 0.0001; q < 0.001, respectively)*, Col9a2* (*P* < 0.0001; q < 0.004 and *P* < 0.0003; q < 0.004 respectively)*, Col9a3* (*P* < 0.0004; q < 0.015 and *P* < 0.0001; q < 0.001, respectively)*, Col11a2* (*P* < 0.0001; q < 0.004, and *P* < 0.0001; q < 0.001, respectively)*, Col16a1* (*P* < 0.0031; q < 0.05 and *P* < 0.0001; q < 0.001, respectively)*,* *Col18a1* (*P* < 0.0001; q < 0.004 and *P* < 0.0001; q < 0.001, respectively), *Col22a1* (*P* < 0.0034 and *P* < 0.0041; q < 0.025, respectively*), Col23a1* (*P* < 0.0031 and *P* < 0.0099; q < 0.048, respectively); *Col24a1* (*P* < 0.012 and *P* < 0.0033; q < 0.0212, respectively), *Col27a1* (*P* < 0.0027; q < 0.05 and *P* < 0.001; q < 0.009). Expression of *Col1a2* gene was upregulated in the winners (*P* < 0,043), and down regulated in the losers (*P* < 0.0004; q < 0.005). In comparison with the controls, the winners expression rate was increased in the *Col1a1* (*P* < 0.005), *Col4a5* (*P* < 0.005), *Col4a6* (*P* < 0.017), *Col5a1* (*P* < 0.017), *Col6a1* (*P* < 0.043), *Col6a2* (P < 0.008), *Col6a4* (P < 0.009) genes. In the losers, expression of the *Col4a1* (*P* < 0.024), *Col6a3* (*P* < 0.001, q < 0.001), *Col11a1* (*P* < 0.005, q < 0.029), *Col15a1* (*P* < 0.047) *Col26a1* (*P* < 0.009, q < 0.042) genes were increased and the *Col3a1* (*P* < 0.0006~~1~~, q < 0.006) and *Col13a1* (*P* < 0.046) genes were decreased. In the losers increased expression of the *Plod1* (*P* < 0.008; q < 0.041 and *Plod3 (P* < 0.0011; q < 0.010 genes as well as decreased expression of *Plod2* gene (*P* < 0.0002; q < 0.003 в was found. In the winners increased expression of the *Pcolce (P* < 0.006) and *Plod3* (*P* < 0.008) genes was found.

**In the midbrain raphe nuclei** in the winners and losers expression of the *Col6a3* (*P* < 0.002 and *P* < 0.009, respectively)*, Col9a2* (*P* < 0.006 and *P* < 0.018, respectively)*,* genes were increased and *Col6a2* (*P* < 0.005 and *P* < 0.049, respectively)*,* *Col15a1* (*P* < 0.004 and *P* < 0.014, respectively)*, Col24a1* (for both *P* < 0.0001; q < 0,005)*, Col25a1* (*P* < 0.039 and *P* < 0.001, respectively) genes were decreased (Figure 2, Supplement Table 2, 4*)*. Additionally, in the losers expression of the *Col6a1* (*P* < 0.003)*, Ccbe1 (P* <0.008*)* and *Colq* (*P* < 0.007) genes were upregulated. In the winners decreased expression of the *Plod1* (*P* < 0.025) and *Plod3 (P* < 0.013) and increased expression of *Plod2* (*P* < 0.0095) genes was found.

**In the VTA** of the winners and losers decreased expression of the *Col1a2* (*P* < 0.022 and *P* < 0.001, respectively), *Col5a2* (*P* < 0.014 and *P* < 0.002, respectively), *Col5a3* (*P* < 0.006 and *P* < 0.032, respectively), *Col11a2* (*P* < 0.001 and *P* < 0.012, respectively), *Col24a1*(for both *P* < 0.013), *Colq* (*P* < 0.0004 and *P* < 0.0001; q < 0.007, respectively) genes were common for both social groups. The *Col1a1* (*P* < 0.016), *Col4a1* (*P* < 0.035), *Col9a2* (*P* < 0.015), *Col9a3* (*P* < 0.002), *Col16a1* (*P* < 0.007), *Col23a1* (*P* < 0.004) in the winners, and *Col27a1* (*P* < 0.013) in the losers specifically decreased their expression. In the losers the *Col25a1* (*P* < 0.001; q < 0.021) and *Colgalt2* (*P* < 0.021) genes were upregulated.

**In the hippocampus** there are no genes that changed their expression in the same direction common for both social groups. However the increased expression of the *Col1a1* (*P* < 0.0001; q < 0,005), *Col1a2* (*P* < 0.0005; q < 0,029), *Col3a1* (*P* < 0.0001; q < 0,005), *Col5a1* (*P* < 0.0001; q < 0,005), *Col5a2* (*P* < 0.0005; q < 0,031), *Col6a1* (*P* < 0.0001; q < 0,005), *Col6a2* (*P* < 0.0001; q < 0,005), *Col9a2* (*P* < 0.015), *Col12a1* (*P* ≤ 0.007), *Col13a1* (*P* < 0.033), *Col18a1* (*P* < 0.0053), *Col23a1* (*P* < 0.001; q < 0,046), *Col27a1* (*P* ≤ 0.0003; q < 0,021) was revealed in the losers and *Col6a3* (*P* < 0.0001; q < 0,021) in the winners. The *Col6a4* gene were upregulated in the winners (*P* < 0.0004) and downregulated in the losers (*P* < 0.0001; q < 0,01). Oppositely, the *Col16a1* gene were downregulated in the winners (*P* < 0.014) and upregulated in the losers (*P* < 0.0001; q < 0,009). The *Col8a2* (*P* < 0.021) and *Col11a2* (*P* < 0.034) genes were specifically downregulated in the winners. Expression of the *Plod2* gene was increased in the losers (*P* < 0.036).

**In the striatum** the *Col9a2* and *Col27a1* genes were upregulated both in the winners (*P* < 0.036 and *P* < 0.048) and losers (*P* < 0.024 and *P* < 0.001, q < 0.041). Specifically the *Col11a2* (*P* < 0.02) and *Col23a1* (*P* < 0.002) genes in the winners and the *Col1a1* (*P* < 0.0001, q < 0.002), *Col1a2* (*P* < 0.0001, q <0.002), *Col3a1* (*P* < 0.0003, q < 0.015), *Col5a1* (*P* < 0.0001, q < 0.005), *Col5a3 (P* < 0.027), *Col8a1* (*P* < 0.0001, q < 0.004), *Col8a2* (*P* < 0.0001, q < 0.004), *Col9a3* (*P* < 0.0001, q < 0.004), *Col12a1* (*P* < 0.001, q < 0.04), *Col15a1* (*P* < 0.027), *Col16a1*(*P* < 0.009), *Col18a1* (*P* < 0.0001, q < 0.004), *Col24a1* (*P* < 0.0001, q < 0.004), *Col25a1* (*P* < 0.039), *Colq* (*P* < 0.0011, q < 0,043) genes in the losers were upregulated and the *Col6a1* (*P* < 0.001, ~~q ≤ 0.05~~) and the *Col6a5* (*P* < 0.025) were downregulated. The *Col6a4* gene was upregulated in the winners (*P* < 0.003) and was downregulated in the losers (*P* < 0.001, q < 0.043). In the losers we found increased expression of the *Cthrc1* (*P* < 0.0001, q < 0.004, *Plod1* (*P* < 0.007), *Pcolce* (*P* < 0.0001, q < 0.004), and *Pcolce2* (*P* < 0.004) genes.

| **S1 Table 2.** Differentially expressed *Col* genes in brain regions of male mice with repeated experience of agonistic interactions | | | |
| --- | --- | --- | --- |
| **Winners** | | **Losers** | |
| **Hypothalamus** | | | |
| *Col1a1,* ***Col1a2****,* ***Col4a2****, Col4a5, Col4a6, Col5a1,* ***Col5a3,*** *Col6a1, Col6a2, Col6a4,* ***Col9a2, Col9a3, Col11a2****,****Col16a1****,* ***Col18a1,*** ***Col22a1, Col23a1****,* ***Col24a1****,* ***Col27a1****, Pcolce,* ***Plod3*** | up | *Col4a1,* ***Col4a2,*** ***Col5a3,*** *Col6a3,* ***Col9a2,*** ***Col9a3,*** *Col11a1,* ***Col11a2,*** *Col15a1,* ***Col16a1****,* ***Col18a1, Col22a1, Col23a1****,* ***Col24a1****, Col26a1,* ***Col27a1,*** *Plod1,* ***Plod3***  ***Col1a2****, Col3a1, Col13a1, Plod2* | up  down |
| **Midbrain Raphe Nuclei** | | | |
| ***Col6a3****,* ***Col9a2****, Plod2*  ***Col6a2 Col15a1, Col24a1, Col25a1,*** *Plod1, Plod3* | up  down | *Col6a1,* ***Col6a3, Col9a2****, Colq, Ccbe1*  ***Col6a2, Col15a1, Col24a1, Col25a1*** | up  down |
| **Hippocampus** | | | |
| *Col6a3,* ***Col6a4***  *Col8a2, Col11a2,* ***Col16a1*** | up  down | *Col1a1, Col1a2, Col3a1, Col5a1, Col5a2, Col6a1, Col6a2, Col9a2, Col12a1, Col13a1,* ***Col16a1,*** *Col18a1, Col23a1, Col27a1, Plod2*  ***Col6a4*** | up  down |
| **Striatum** | | | |
| ***Col6a4,*** ***Col9a2,*** *Col11a2, Col23a1,* ***Col27a1*** | up | *Col1a1, Col1a2, Col3a1, Col5a1, Col5a3, Col8a1, Col8a2,* ***Col9a2,*** *Col9a3, Col12a1, Col15a1, Col16a1, Col18a1, Col24a1, Col25a1,* ***Col27a1,*** *Colq, Cthrc1, Plod1, Pcolce, Pcolce2*  *Col6a1,* ***Col6a4,*** *Col6a5* | up  down |
| **Ventral tegmental area** | | | |
| *Col1a1,* ***Col1a2,*** *Col4a1,* ***Col5a2,*** ***Col5a3,*** *Col9a2, Col9a3,* ***Col11a2,*** *Col16a1, Col23a1,* ***Col24a1, Colq*** | down | *Col25a1, Colgalt2*  ***Col1a2,*** ***Col5a2,*** ***Col5a3,*** ***Col11a2, Col24a1,*** *Col27a1,* ***Colq*** | up  down |
| Up – upregulation; down – downregulation; bold – gene expression is changed in the winners and losers in brain regions | | | |

| **S1 Table 3.** Differentially expressed *Col* genes in the hippocampus, striatum and hypothalamus of male mice with repeated experience of agonistic interactions | | | | | | | | | | |
| --- | --- | --- | --- | --- | --- | --- | --- | --- | --- | --- |
| Official gene symbol | **Hippocampus** | |  | Official gene symbol | **Striatum** | |  | Official gene symbol | **Hypothalamus** | |
|  | Winners | Losers |  |  | Winners | Losers |  |  | Winners | Losers |
| *Col1a1* |  | ΔΔΔΔ* |  | *Col1a1* |  | ΔΔΔΔ* |  | *Col1a1* | ΔΔ |  |
| *Col1a2* |  | ΔΔΔ* |  | *Col1a2* |  | ΔΔΔΔ* |  | *Col1a2* | Δ | ▼▼▼* |
| *Col3a1* |  | ΔΔΔΔ* |  | *Col3a1* |  | ΔΔΔ* |  | *Col3a1* |  | ▼▼▼* |
| *Col5a1* |  | ΔΔΔΔ* |  | *Col5a1* |  | ΔΔΔΔ* |  | *Col4a1* |  | Δ |
| *Col5a2* |  | ΔΔΔ* |  | *Col5a3* |  | Δ |  | *Col4a2* | ΔΔ | ΔΔΔΔ* |
| *Col6a1* |  | ΔΔΔΔ* |  | *Col6a1* |  | ▼▼ |  | *Col4a5* | ΔΔ |  |
| *Col6a2* |  | ΔΔΔΔ* |  | *Col6a4* | ΔΔ | ▼▼▼* |  | *Col4a6* | Δ |  |
| *Col6a3* | ΔΔΔΔ* |  |  | *Col6a5* |  | ▼ |  | *Col5a1* | Δ |  |
| *Col6a4* | ΔΔΔ | ▼▼▼▼* |  | *Col8a1* |  | ΔΔΔΔ* |  | *Col5a3* | ΔΔ* | ΔΔΔΔ* |
| *Col8a2* | ▼ |  |  | *Col8a2* |  | ΔΔΔΔ* |  | *Col6a1* | Δ |  |
| *Col9a2* |  | Δ |  | *Col9a2* | Δ | Δ |  | *Col6a2* | ΔΔ |  |
| *Col11a2* | ▼ |  |  | *Col9a3* |  | ΔΔΔΔ* |  | *Col6a3* |  | ΔΔΔ* |
| *Col12a1* |  | ΔΔ |  | *Col11a2* | ΔΔ |  |  | *Col6a4* | ΔΔ |  |
| *Col13a1* |  | Δ |  | *Col12a1* |  | ΔΔΔ* |  | *Col9a2* | ΔΔΔΔ * | ΔΔΔ* |
| *Col16a1* | ▼ | ΔΔΔΔ* |  | *Col15a1* |  | Δ |  | *Col9a3* | ΔΔΔ * | ΔΔΔΔ* |
| *Col18a1* |  | ΔΔ |  | *Col16a1* |  | ΔΔ |  | *Col11a1* |  | ΔΔ* |
| *Col23a1* |  | ΔΔΔ* |  | *Col18a1* |  | ΔΔΔΔ* |  | *Col11a2* | ΔΔΔΔ * | ΔΔΔΔ* |
| *Col27a1*  *Plod2* |  | ΔΔΔ*  Δ |  | *Col23a1* | ΔΔ |  |  | *Col13a1* |  | ▼ |
|  | | |  | *Col24a1* |  | ΔΔΔΔ* |  | *Col15a1* |  | Δ |
|  |  |  |  | *Col25a1* |  | Δ |  | *Col16a1* | ΔΔ * | ΔΔΔΔ* |
|  |  |  |  | *Col27a1* | Δ | ΔΔΔ* |  | *Col18a1* | ΔΔΔΔ * | ΔΔΔΔ* |
|  |  |  |  | *Colq* |  | ΔΔΔ* |  | *Col22a1* | ΔΔ | ΔΔ* |
|  |  |  |  | *Cthrc1* |  | ΔΔΔΔ* |  | *Col23a1* | ΔΔ | ΔΔ* |
|  |  |  |  | *Plod1* |  | ΔΔ |  | *Col24a1* | Δ | ΔΔ* |
|  |  |  |  | *Pcolce* |  | ΔΔΔΔ* |  | *Col26a1* |  | ΔΔ* |
|  |  |  |  | *Pcolce2* |  | ΔΔ |  | *Col27a1* | ΔΔ* | ΔΔΔ* |
|  |  |  |  |  | | |  | *Plod1* |  | ΔΔ |
|  |  |  |  |  |  |  |  | *Plod2* |  | ▼▼▼ |
|  |  |  |  |  |  |  |  | *Plod3* | ΔΔ | ΔΔ |
|  |  |  |  |  |  |  |  | *Pcolce* | ΔΔ |  |
| Note: Grey box - genes with opposite changes in the winners and losers  Δ – increase: Δ - *P* < 0.05; ΔΔ - *P* < 0.01; ΔΔΔ - *P* <0.001; ΔΔΔΔ - *P* < 0.0001; *- q < 0.05. ▼- decrease: ▼- *P* < 0.05; ▼▼- *P* < 0.01; ▼▼▼- *P* < 0.001; ▼▼▼▼- *P* < 0.0001; * - q<0.05. | | | | | | | | | | |

| **S1 Table 4.** Differentially expressed *Col* genes in the midbrain raphe nuclei and VTA in male mice with repeated experience of agonistic interactions | | | | | | |
| --- | --- | --- | --- | --- | --- | --- |
| Official gene symbol | **Midbrain raphe nuclei** | |  | Official  gene  symbol | **VTA** | |
|  | Winners | Losers |  |  | Winners | Losers |
| *Col6a1* |  | Δ Δ |  | *Col1a1* | ▼ |  |
| *Col6a2* | ▼▼ | ▼ |  | *Col1a2* | ▼ | ▼▼▼ |
| *Col6a3* | Δ Δ | Δ Δ |  | *Col4a1* | ▼ |  |
| *Col9a2* | Δ Δ | Δ |  | *Col5a2* | ▼ | ▼▼ |
| *Col15a1* | ▼▼ | ▼ |  | *Col5a3* | ▼▼ | ▼ |
| *Col24a1* | ▼▼▼▼* | ▼▼▼▼* |  | *Col9a2* | ▼ |  |
| *Col25a1* | ▼ | ▼▼▼ |  | *Col9a3* | ▼▼ |  |
| *Colq* |  | Δ Δ |  | *Col11a2* | ▼▼▼ | ▼ |
| *Ccbe1* |  | Δ Δ |  | *Col16a1* | ▼▼ |  |
| *Plod1* | ▼ |  |  | *Col23a1* | ▼▼ |  |
| *Plod2* | Δ Δ |  |  | *Col24a1* | ▼ | ▼ |
| *Plod3* | ▼ |  |  | *Col25a1* |  | ΔΔΔ * |
|  | | |  | *Col27a1* |  | ▼ |
|  |  |  |  | *Colgalt2* |  | Δ |
|  |  |  |  | *Colq* | ▼▼▼ | ▼▼▼▼* |
| Δ – increase: Δ *- P* < 0.05; ΔΔ - *P* < 0.01; ΔΔΔ - *P* < 0.001; *- q < 0.05. ▼- decrease: ▼- *P* < 0.05; ▼▼- *P* < 0.01; ▼▼▼- *P* < 0.001; ▼▼▼▼- *P* < 0.0001; *- q < 0.05. | | | | | | |

| **S1 Table 5.** Overlapping and specific differentially expressed genes in brain regions of the winners with repeated experience of aggression | |
| --- | --- |
| **Brain regions, winners** | **Genes** |
| Hpt-MRN-Str-VTA | *Col9a2* |
| Hpt- Str-Hpc-VTA | *Col11a2* |
| Hpt-MRN-VTA | *Col24a1* |
| Hpt- Hpc-Str | *Col6a4* |
| Hpt-VTA-HpC | *Col16a1* |
| Hpt-Str-VTA | *Col23a1* |
| Hpt-MRN | *Col6a2, Plod3* |
| Hpt-Str | *Col27a1* |
| Hpt-VTA | *Col1a1, Col1a2, Col5a3, Col9a3* |
| Hpc-MRN | *Col6a3* |
| HpC | *Col8a2* |
| MRN | *Col15a1, Col25a1, Plod1* |
| VTA | *Col4a1, Col5a2, Colq* |
| Hpt | *Col4a2, Col4a5, Col4a6, Col5a1, Col6a1, Col18a1, Col22a1* |
| Note: MRN – midbrain raphe nuclei; HPC – hippocampus; VTA – ventral tegmental area; STR – striatum; HPT – hypothalamus. | |

| **S1 Table 6.** Overlapping and specific differentially expressed genes in brain regions of the losers with repeated experience of defeats | |
| --- | --- |
| **Brain regions** | **Genes** |
| HPC-HPT-MRN-STR | *Col9a2* |
| HPC-HPT-STR-VTA | *Col1a2, Col27a1* |
| HPT-MRN-STR-VTA | *Col24a1* |
| HPC-HPT-STR | *Col3a1, Col16a1, Col18a1* |
| HPC-MRN-STR | *Col6a1* |
| HPT-MRN-STR | *Col15a1* |
| HPT-STR-VTA | *Col5a3* |
| MRN-STR-VTA | *Col25a1, Colq* |
| HPC-HPT | *Col13a1, Col23a1, Plod2* |
| HPC-STR | *Col1a1, Col5a1, Col6a4, Col12a1* |
| HPC-MRN | *Col6a2* |
| HPC-VTA | *Col5a2* |
| HPT-STR | *Col9a3, Plod1* |
| HPT-MRN | *Col6a3* |
| HPT-VTA | *Col11a2* |
| HPT | *Col4a1, Col4a2, Col11a1, Col22a1, Col26a1, Plod3* |
| STR | *Col6a5, Col8a1, Col8a2, Cthrc1, Pcolce, Pcolce2* |
| MRN | *Ccbe1* |
| VTA | *Colgalt2* |
| Note: MRN – midbrain raphe nuclei; HPC – hippocampus; VTA – ventral tegmental area; STR – striatum; HPT – hypothalamus. | |

| **S1 Table 7.** Comparison of *Col** gene expression (in FPKM units) in the winners *vs* losers | | | | |
| --- | --- | --- | --- | --- |
| **HPC** | Winners  *vs* losers |  | **STR** | Winners  *vs* losers |
| *Col1a1* | Δ Δ Δ Δ* |  | *Col1a1* | Δ Δ Δ* |
| *Col1a2* | Δ Δ* |  | *Col1a2* | Δ Δ Δ Δ* |
| *Col3a1* | Δ Δ Δ Δ* |  | *Col3a1* | Δ Δ |
| *Col4a1* | Δ Δ |  | *Col5a1* | Δ Δ Δ* |
| *Col4a2* | Δ Δ |  | *Col6a1* | ▼▼ |
| *Col5a1* | Δ Δ Δ Δ* |  | *Col6a2* | ▼ |
| *Col5a2* | Δ Δ |  | *Col6a4* | ▼▼▼▼* |
| *Col6a1* | Δ Δ Δ Δ* |  | *Col6a5* | ▼ |
| *Col6a2* | Δ Δ Δ Δ* |  | *Col8a1* | Δ Δ Δ Δ* |
| *Col6a3* | ▼▼▼▼* |  | *Col8a2* | Δ Δ Δ Δ* |
| *Col6a4* | ▼▼▼▼* |  | *Col9a3* | Δ Δ Δ Δ* |
| *Col8a1* | Δ |  | *Col12a1* | Δ Δ Δ |
| *Col8a2* | Δ |  | *Col18a1* | Δ Δ Δ Δ* |
| *Col9a2* | Δ Δ Δ* |  | *Col24a1* | Δ Δ |
| *Col9a3* | Δ |  | *Col25a1* | Δ |
| *Col11a2* | Δ Δ |  | *Pcolce* | Δ Δ Δ Δ* |
| *Col12a1* | Δ |  | *Colq* | Δ Δ |
| *Col13a1* | Δ |  |  |  |
| *Col16a1* | Δ Δ Δ Δ* |  |  |  |
| *Col18a1* | Δ Δ* |  | **MRN** |  |
| *Col23a1* | Δ |  | *Col5a2* | Δ |
| *Col27a1* | Δ Δ Δ* |  | *Col27a1* | ▼ |
|  |  |  |  |  |
| **HPT** |  |  | **VTA** |  |
| *Col5a1* | ▼ |  | *Col6a1* | Δ Δ |
| *Col4a6* | ▼▼ |  | *Col9a2* | Δ |
| *Col6a4* | ▼▼▼ |  | *Col15a1* | Δ Δ |
| *Col13a1* | ▼▼▼ |  | *Col18a1* | Δ Δ Δ Δ* |
| *Col16a1* | Δ |  | *Col25a1* | Δ Δ Δ Δ* |
|  |  |  | *Col27a1* | ▼▼▼▼* |
| Note: MRN – midbrain raphe nuclei; HPC – hippocampus; VTA – ventral tegmental area; STR – striatum; HPT – hypothalamus. Δ – increase: Δ - *P* < 0.05; ΔΔ - *P* < 0.01; ΔΔΔ - *P* < 0.001; ΔΔΔΔ - *P* < 0.0001; *- q < 0.05.  ▼- decrease: ▼ - *P* < 0.05; ▼▼- *P* < 0.01; ▼▼▼- *P* < 0.001; ▼▼▼▼- *P* < 0.0001;*- q < 0.05. | | | | |

Comparison of gene expression in the winners *vs* losers revealed maximal number of genes with different expression in the hippocampus and striatum and minimal number of genes with different expression in the hypothalamus.

Similar changes in the winners and losers in these brain regions can be associated with a general reaction to stress common for both participant of social conflicts

The midbrain raphe nuclei and ventral tegmental area contain the body of serotonergic and dopaminergic neurons, respectively. We assume that these brain regions have partly similar molecular mechanisms of neuronal regulations.
